# Supplementary material for: Identification of novel genes responsible for a pollen killer present in local natural populations of Arabidopsis thaliana
Source: PLoS Genet. 2025 Jan 13;21(1):e1011451. doi: 10.1371/journal.pgen.1011451 (PMC11761171; doi:10.1371/journal.pgen.1011451)
Supplement: S1 File — (DOCX) [file pgen.1011451.s001.docx]

**File S: Sequence of the Mr-0 insertion in *AT3G62610*.**

Genomic sequence (10,946 bp) from ATG to Stop of *AT3G62610* with the large insertion present in the second intron. The sequence missing in Bur-0 (from 2,280 bp to 4,503 bp) is indicated between brackets. The two parts of *AT3G62610*, at the beginning and at the end of the sequence, are highlighted in dark grey. *APOK3-like* (*KPOK3C*) is highlighted in light grey, the 5’-UTR determined by 5’-RACE is in lower case and italics, and the region homologous to the antidote gene *APOK3* is underlined. The black arrow indicates the 5’‑end of the *APOK3-like* transcript predicted by EuGene. In both *AT3G62610* and *APOK3-like*, introns are in lower case. Primers used are written in blue, with their names and orientations below the sequence. The two guide-RNAs used for CRISPR-Cas9 mutagenesis of *APOK3-like*, gRNA_APOK3-L#1 and gRNA_APOK3-L#2, are highlighted in red.

ATGGGAAGGGCGCCGTGCTGTGAGAAGGTAGGAATGAAGAAGGGGCGCTGGACGGCGGAGGAAGACCGGACTCTCTCCGACTACATTCAGTCCAACGGCG 100

AAGGATCATGGCGTTCTCTTCCCAAAAATGCCGgtaaagttttttttaatctatacagtactctgtctctcgactatcgcatgcatctcataagatagtt 200

tttttgtttggtcaaaatttataagagaatctatatgcatcgagaagttaattaatcttacgtacggttcaatctatatgcataatattttaaagtacat 300

aaagtgtttattgataattcagaccaaattgccttcttgttttatttacatgtatatatggactgtaacatcacaaataaaacgataatttatttcaata 400

tttaggaaaagatgagtaatcgctgcaggctttattatgccttaattaaaatctgacgtacattttaaaaagGGCTAAAGAGATGTGGAAAGAGCTGTAG 500

ATTGAGATGGATAAACTATTTGAGATCAGACATCAAGAGAGGAAACATAACTCCCGAAGAAGAGGACGTCATTGTTAAACTGCATTCCACTTTGGGAACC 600

----AT3G62610_F1--

AGgtaactttttcatttgaacaaaagtttattaatgtcgatatatttccaatagaaaaagtaTCAAGGAGAAGGCTCAGTCAACTGAAACAGAGCTCAGG 700

->

TCAAACCTTCTGCTCAGCTCAGAGACTTCTCAACGCAAGGTTATGGGCCAAGGTATTCAGCCCAGCAACATTCAGAAAACTACAGAGCCCACCAAGCCCA 800

ATAACAGACAGAGTACATCTCTGCAGCTTCACGACAAAGAGAACAATGGCGTCAAAGAAGGAACCGTTGCAAAAGCAAAACGAGTGAGCTGTGCATCCGT 900

ATCAAAGATAAAGACGAAGAATCGATATGACGTTCTTAGCCTCCTCTCATCAAATGAAATGTGCGAAGGCTCCTAGGGTTTTCACAAGTGTATAAATAAA 1000

GAGACAGAGTCTCATTGTAAAAGTAAGAGAGAAGAGCTTAACAAAGTTTCAGTATTTCTCCTTCACTTTCAAATCTTTACATCCCCTACCCTTGAGTGAT 1100

TGAGTTTGAGATATGTTGCGACTAGGACTTCGGAGCATGAGTAAGGCAGTGGGAAGCTCAAGCTCCAATGTTCAAAGAAGGCCTTCATCAGTTTTCCCAA 1200

CTTTAGACCTCAAGAACATTACTCGTTCCACTTTCCTCAACCCTATGTTTTCTTCACAAGCTTTGAACAGAATTCCACTTGCTCTCCAGGTATTTGTTCA 1300

CTTCTTAGCTTTGCTCTAGCTTTTTTCTTCTTGACTCGTTCTTGTTCTCTTTAATGAACCATTTTGGCTTGTTCACTTAATAAACCAAACTTTCTCAAAT 1400

CTCAAACTCAAACCTGCAACTTTCACAGCATGAGTTCTGAACCTGTTTCTTCACCCATCTGCAACTCTTCTTGAATTATGAAACCTTGACCCCCAGAGCC 1500

GTGTGTTAACTAGTATCACAAGCGATTAAAAAATAGATACCTCAAATCATAGAAAAAACATCTACAAAAATAAATAACATCTTCTTTATTCAATGCATTC 1600

CATTTTTCTAGTTTAACATATGTTTTTTTTCTCTTTGACACTCATCTATATTCGCATCCACTCCATCATCTTCAAGAATGTCATGTTCTTTACCATTAGT 1700

TTCCACAAATTCATCAAAATTTCAAGTTTTTAAAAATTTAATAAAGCATTCTCTTAAGATTTTTGCTTGTTCATTTTTTAGTTTTCTCTTTTATTTTTTT 1800

TTGTTTACGTGGATGATATTTTTTCACATTTTCTGTAGTAAATAAAATAAAATAATTATTGTTTCCAATTTTGGAAAAGTTTGAATGAAAAACTTCACTA 1900

ATTTTAATTTAATAGAAACGATTTGGTGAAAGTACGTTTAAATAAATAAATAAAATGTATTACCTTAGAATCAAAAGTTTGACCAAAGACAAAAACTCAG 2000

GAAAACATAACAACGAAAATGAAAGCCAACAAATCTCTTTGCCTAATTGTTAATTAACTAATATTATGTGCCCACTTTTTAAAAGTTAATTACTATTTTA 2100

GAAGTCCATGTTTGAAATTAGATTATAGAAGCCCTATTTCAGTTTTCTTTATATTTTTAAGTTTTAATGTTGTATAACACTTATATTTATAAAATTTAAT 2200

TGAGTTAACATGACACTAATATATATATGCCCCTAATTGTTTTTCTAAAATCACATGCCCTAAACGAAGGCTTCTTTTT**[**TCCTC*actgtagtacgtctct* 2300

------Mr530B_F6------> ----Mr530B_F5------

*gttacttctctatcctcactgagtttctctgttttttcttttgcagcgttgcaaaacaagtgtgatctgggacacggagaacgttcccattcctaattcg* 2400

>

*gtggatccttgtttcttgtcaagcaatatt***ATG**CGTTCCCTTCGAAAATGCAATTTTTTTGGACCTGTGACGATCATCGCCGTCGGAAACTTCGAAAACT 2500

-----Mr530B_F4------>

TTAAAAAAGGAGTTGAAGAGATTCTACATTTAACCGGCGTGGAGATGGATTACGTGAAGCCCCTCAAGATTCAGAGAAAAACGATGAGCGAGAAGAGTGA 2600

CAAGAGGATCTTAGTTAGGATATCACTGTGGATCCAGAAGAATCTTGTCCCAACTAATATCCTCCTTGTAACTGGCGATGGAGGATTCGCTGATGGAATG 2700

AATGAATGGGGATTGTTGGGTTTCAATTTTCTTATAGCCCACCCGGATTTGAGAAGTCATTCATATAAGTTGGAGTTTATGGCAACAACGATATGGGCTT 2800

<----Mr530B_R3------

GGTCAACCCTAGCAGCGGGAGGATTGCCAACCACATACCATCAGACTATTTACCACGACGTACGCAAACGGAATAGATACGCTAATGGACCAGTCACTCG 2900

<--gRNA_APOK3-L#1---

TGTCTTGGAAACCATTGACCAATTGGAAGCACAAGGTATTACGGCCACTGAAGGTAATATTTGGACTTTTATCCAACAGAACTATCGCCGAGCTTGTATT 3000

-----Mr530B_F8----->

<----Mr530B_R2------ <----Mr530B_R1------

AATTTAGGAAAGTCGCTGAACATTGCCGTGAAGATCAACAAAGTCACAAAGCATGTCAATGAGGAGGATGGTGCAACTTTCTATGTTACTCGAAGGGGGG 3100

<----Mr530B_R0------

CAATCGATAGTTCTGCCCCTGCTGAATCAAAAGCATATCCAAAATCAGCTAATATCAGTTCAATCCATAGTATGAGgtaactaactcaaattacttgtag 3200

ataacttactaatttttgaaaggtttattgattaaaatcaactatagGGAATCTCTTCTGGAGGAAACAGAGGAAGACTACTACAGACGCTTGGCACTTT 3300

---gRNA_APOK3-L#2-->

<----Mr530B_R5----

TTGCCCTGAGGAATCATGGTGGAGAGGATGCCATAAATGTTATTATAGAATCTCTAGGTGTTGAGAGTTCTATGATTCGCATTGAGgtcaatcaaatttt 3400

--

tatgtatatattgatgactttgagtttgtctcattgttctctaactcaaaatgcattttgtttttgcttcttcttagGCTGCATTTGTGTTGGGACAATT 3500

GGAATCCAAAACAGCTATAGCTTCTCTAAGCAAGATCTTGAGAGATGTGAAGGAGCACCCCATGGTTCGAGTAGAGGCTGCAAAAGCCCTTGGTTTCATT 3600

GCAGgtatattcctagaagctaccacaattttgtgtctttaattaattactctttgacattctttgggttttttttcagATGAGAAGAGCAGAGAAGTAC 3700

TCCAAGAACTTTCAGGCGACCTCGATCCCATTATCGCTAAAGGTTGTGATTCTTCATTGAGTATCTTGGAATTTAAGAATTCAAAAAAATATGACCCTCT 3800

CATATAAAATGGTGTGTGTGGGTCTGTTGTGTAATGAAAACATCTACGTTTCATCTTCCTTTTGTTAAACTTTGTTTTGTATTCAAATGATTTGCAGACG 3900

GTTTAAGCTCTGTTTTAAGCTATTATCTCGGCCTACTTGCATTTCTTCACTTTCTCAGTGAAAGAATGATTAATTTTTTTCATTAGAGTGCACAATTGTT 4000

<-------530_R1------

AATTAATATTTTCTTGTTATAGAAGCAAATTACAAAAAAGCTATCTGACAGATTCCAATGTGATCAGTTCAGTCCAAGCTGTGCTAGTGAAGTCATATTC 4100

TTGATTCAAGTAAGAGTTGTGGCTCGAAAACCAACCATTTGCAGATAAGCTTTCTCCGAGTGAGTGAGTTGAATGATCCAGTTACCCGCCCCCAAGTTAT 4200

CAATCTTCCATGAATCTCTGTTTGCATCTCCTTCTAGTCATCTGAGCCCATCTCGCAAAGATCTCTGCTGCAGCAAACTTACCTGAGAACTTAGCTCTCT 4300

GTGCCAATTCCTCAAATATCTCCGGTTGTCCCCCTCTGTGGAAATCAGCTGCAAACTTGTTATAATCAATCAAAGGGGTTTTCATTCCTTTGTCAAGCAT 4400

CTCCTCCAAGTATCTGCATGCCTCTCTCGACTTCCCTTCACTTATAAGCCCTCTGATCAAAACCGTGTAGGAGTTATCGTCAGGACAAATCCCTTTCTTG 4500

ATC**]**ATCTCGTCCCAAACTGCTCTCCCCATCTCATAGTTTCTCGCCACGAAGTAAGATTTCATTATCATGTTGAACGTGTGTATCGATGGCTCAATTTCGT 4600

TCTGGATCATCTTGTTGTAGATCCTTGTGCCATGTTCCGGCATTTTCTGGTTCGCCATCAGTTTGATCAACGCATTATACGTTTTGCCATCCGGAGGGTG 4700

TCCCTTTTCTTGCATCTCCTTGAGCAGTTCGTAAACAGTATCCAGCTTCTTCTGAGTCCCAAACCCCGTGATCAGACACGTGTAAACCGCAGCATCAGGT 4800

TGCAACCCGGAATCAACCATGTCATCAAAGTACTCAATGGCAGTTTCCATGCTCGACTGTTTGCAGAAGTCCCGGATCATGATGGTATAGCTACGGACAT 4900

TAGGGCAAGGTCCCTTGGATTTCATAACATGGAACAATTTGATCGCATCTGATTTTTTCATACTCCTCAACAACCCTTCAAGCATTACGTTGTGAGCTAC 5000

AATGTCAGGTTTCAATCCGTGATCAATCATATCATTCCAGATTCTAGCTGCTTCTATCAAGTTCCTCACCCTGCACCAACCATTGAGTAGAACAGTGTAA 5100

GTCATCATATTAGGTGTAAACCTCTCCTTCAGCTTATCAAACAGAACCTGAGCTTCTTTTCCAAGCTTAGCCCTGCCAAGACTATCAAGCAAGCAATTAA 5200

TGGTTTCAACACCAATCTTAAACTTGTACTTCTTCATCAACTCAAAGATACCAACAGCTTTCTTCCTCTCTTTCGCAGCAGCGAAAGCTTTCATCGCGAT 5300

AGTGAATGTCTCCATCGTCAATAGACCTTTCGTACCCATCTCTTCGAGTACCGAAACCATCGTCTCGAACTGTCTAGTCTTTGCTAAAATACTCATCATG 5400

GAGTTATATGTTCTTGAGTCATGAGCAAATCCTTGTCTCTCTGCAGCCCAACAGAAGAACCTAAATGCAGGCTTCCTAGCATGTCGAAAACGTTCTAAAA 5500

CCTCAACGATGAGATCATGTGATAAATCTAGTTTCATTTCATCAAGAACAGCTTCCATATTTCTATCTAACGCAAACAATTCATCAATCACTTTGCAGAC 5600

TCTCTCTACTTCTTCCGGATTAGTACTTGATTCAACACAACTGACCCCAGTTTCTTCATCATTATCGCATTCAGATTCAACTTCTTCATCACATCCATCA 5700

CTAACATTAGAACTACCACTAGAGAAACCTCTACACCCAAGACTCGCATCAAGCAGTTGTACAGAGGAGTGTGGCAATGGAATTTGACGGTAATAAGTAG 5800

AAGAATGAATCATACGAGAGAAGAGAGCACCGGAAAATCGTACTTCCTCTTCTCCTCTCCCGCCGATTACGCGGCTTTGACTGGAAATGAATTCATCCGA 5900

ATCGAATGAAGAAGAGCAAATGAGGAAGCTTCTTAATGAAGTCTGAGTGGAGCTTCTTCTCCTAGAGAGATGGAGCCATGGAGCAGCAGCCATGGAAGCT 6000

CGACGGCGGTTAGCAATGGCGAAGTCGGTGAAAAGCCACTGATTTACGGCGGAGAGCTCTTCACGTATTTGGGCCTTTGTTTGATTAGAAGCTTTGAGTG 6100

ATAATAATTTTGACCCATTAAAGGCCCATTGATCTGGTTACATATAACCATTGGGTCCACCAGGAGCATTAAAGCGCAAACGCATAATCATCAAAGGTGT 6200

CAATTTCTTTTTTTAGTTGGAAGTTATCTGTATGCATCTTATCTTCGTTTTAGGGTCGCGATAGTTGGAAGCGCGCGTACAGACGCAGCGATTGGACACA 6300

TGTCAGATACGAATCGATTCGGATATTCAATCGCTTTCCAAGATTTAAATCGAATATTCAATTTTTAAGTTCGGAAATTTCGAATTAAGCATCTGCGAAC 6400

GAGTTTCTGCCATTGATTCCGAGGAAACGCTTGGCTCTGATACCAGTGTTAACGTAAACCAGAAATGAATAAACAGAGTTTGAAATCAATAGAACAAGAG 6500

ATGAAAATAGAGTACTCCGATTAACACACTTGATACAACGTTCGTAAAGAGACGCGATATCAACTTCTCACCTACAAGAGACTCGACGAGAGCTCCGGTA 6600

AAACGATGAGGGAAATCTAGAAGTCTCTAGAGAGAGATTACAACCAGATTATTCGATAATGAAAGCATGAGCATAACATGTTGCTCTTATACAAAAAGGT 6700

AAATGTTAATGCACGAATTAAACAAGTACGAAAATGGGATCTCTAGGGAAGGAAGAAGAATCTTTCTATTAATGACGAGCCCGCGACTTAGGCGAATAGA 6800

GTAAGAACGAGCCGATTGTAGATGAAAGACGAGCTTGAGGCCGGAAGACGATCTTGATAGCTGAACCTTGTAAAAGGCTGCCTACGTACCCTTAGAAAGG 6900

GATCAAGCCACACGTAGTTCATATCGCGATAAACGAGCTAGGTCATTACTGGAGGAAGGTCGTATGTCGAGTTAGGCAGTTTTAGGGATGATCCGGCGGA 7000

TAAGTGACGAGTTGAATGGCTTCTCGAGACGTGCGAGTAGGTTAGTTACTTTGGGCGGGCAAGTTGGCGAGACACTTCGGACGGACGAGTAAACGAGCTA 7100

CTCAAGATGGATGGGCAAACGAGGTACTTCGGACGGGTGAGCTGACGAGCTGCTTGAGACGGGCGAACTGATGAGCTACTTGAGACGGACCGGCTGACGA 7200

GATGCTTCAGACGGGTAAGCTGACGAGACGGGCGAACTATGAGCTACTTGAGACGGACCGGCTGGCGAGATATTTCGGACGGGTGAGCTGACGAGCTGCT 7300

TGAGACGGGCGAACGGATGAGCTACTTGAGACGGACCGGCTGGCGAGATACTTCGGACGGGTGAGCTGACGAGCTGCTTGCGACGGGAGAGCTGACGAGC 7400

TACTTGAGACGGACCGGCTGACGAGATGCTTCAGACGGGTAAGCTGACGAGCTGGTTAGACGGGAGAGCTGACGAGCTTCTCTTTGTCTCTAAGTGTCCT 7500

CCTGGAATCCCCTTTGACAAGACATGCTATTCCGTATTTATAGCCTTCTGCGTGTAGGGCATTCCTCCAGGGTTTTGACGAGCCTACCCTCTTGACTTAA 7600

CATTCTGCTTAGGCCTTTGACTTGGATTTGGGCCTTGGGAAAAGCAAGCCCAAATCTAACCCCTAACAGTTGCCCCCCAATCCTGATGCACTGTTGATGA 7700

CGTGGATCAGGATTTCAAGTGCACGAAACTCAATCGAAGGAAGTGAAAAATCGCGCATGGGGAAGACGCAATGTTTAGTTTGTCTTTCATGTTTCTCAAG 7800

GTTCGAGGATCGAAACGTCGCGTGGCTATCAAAGTATAGGCTGCTATTACGGCAATGTGCTGTCTCGGTGATAGTCAAGCGAGACGACAAGGAAGGTTAA 7900

AAGGGTCCCTCGGCTGTTGCGTGACCGTCGTATTTCTGAAGAGCCTCCTGGATCAAAGTGGTCGTCTGGTGTTCGAGTTGGGATGTTCGAGTCATTCGAA 8000

AGTGGTTCTTTGAGGGGGAGAGGGAAGAGACGAGGGTCGTTAATCGTGATGAACAACGAGCTAGCGTTTCGAACTCGTCCGATGCATTTTGCGTGCCAAT 8100

GATCATTATTGGAGTTATCGTTTCGTTTGAAGGAGGCGTTGCTTTTGCTTTGAACTCTGGTGTTTGTGCTGAGCTCGCTGTTTGAGAGGCGAGATTGCTC 8200

AAGCGGTGCCCGATCTAGACGAAATCTTGAGCCGAGGTTGAGAAGATGAGTGGGAAGTATGATAACTTAGTTGAACGCGCTTTTTCAGGGGGAAAAGATC 8300

TTCAGGAGCGTCGGTTGGAGTATGTCGACTTTTTTCGAAAGGTCGGCGGTTGATAAGATGGCGGAGCTCATCGAGAAGCGATTGAATCGGACCAAGACCT 8400

ATTGACGATACGAAGCTGGCGGAGCCAAAATTTTTGAATTCTATCAAGTGTATGGGAACCCTCAAACCCCAAGCGGCGATGAAAGGGGGCGACGAGGGGG 8500

AAAAATAAGACCGAAAGATGCCGGCTTGGATTGGATGCTAACATTTTTGAAGGGGGGGGGACCACCCTTTATTTTTTAGTTTTCGAGCTCGGGGGGTTTT 8600

CCTGTTTAAATTTTTTTTCCCCGGCCCGGTTTGAAATCTTTTTTTTTTTTCTTTTCTTCCGGGGCCAAGAAAAAGGCGGTCCCCCCCAAGGGCGGGAAGG 8700

TCCAAAAAGGCGGGTTGGGGGAAGAAACCTTTATCCGGTTTTTTTTCCAAAAGGGACCCCCGGGGTGGCGGGGGGGATTTAACCCCTTTTCTTTTTTCGG 8800

GTTTTCTTCCAGGTTGGGGTTGGGGGGTTCGGGTTTTTGGGGGGGCCCTTTAAAATGGGGCCCCGATTGATTTTTTTCTTTGATTAAATGGCCAACCCAT 8900

TTTTGGCCCCCTTTATTTTTTCGAAAAGGGGTAAAGGTCGGGGCTTTTCCGGGAATTTTTTGGGGGGAAAGGGGGCGGGAAAATTTTTTAACGGATTCGG 9000

GGGGGGGGGGTTGGCCCACCAAAAAAAATCTAAAAGAAATATTCCCCCCCCCCCCCTTTTTTTGAAAAACAATGCCGGTGTGGAACTCGGTCATTTGAGA 9100

ATTTTTGCGAATCGGAATGGCGGTTGAGTGTGCAAGCGTTGAATGTGCGAATGGTGAGCTCGGCGCTCATTGCGAATGGGCCGCGAGCCTTTGAAGAAGA 9200

AGCCTATGTCAAAGCCCACTTGGATTAAACGGTCTTCTTCTTCATGTGAAGACAAAACCTGGCAAAAGAGACGCAGTGACGTCGGAGACCCGTACGAACT 9300

CAGCTGCATTTACGGTAAAGCTCACCAATCGATTCCTGGCCCTTGATGAAGAAGACTCAAGCGGATGATGCCACCTGTTAAGACACATTAGGGTTTTACT 9400

CTGTTTTGTCTCAAAATCTGAAGACAAAACCTGGCAAAAGAGACGCAGTGACGTCGGAGACCCGTACGAACTCAGCTGCATTTACGGTAAAGCTCACCAA 9500

TCGATTCCTGGCCCTTGATGAAGAAGACTCAAGCGGATGATGCCACCTGTTAAGACACATTAGGGTTTTACTCTGTTTTGTCTCAAAATCTTTCACTTTC 9600

TTTCATTTTCATACACTTTCTCTCCCTCTCTGGCTCTCTCCCCTATCAAGGAGAAGGCTCAGTCAACTGAAACAGAGCTCAGGTCAAACCTTCTGCTCAG 9700

CTCAGAGACTTCTCAACGCAAGGTTATGGGCCAAGGTATTCAGCCCAGCAACATTCAGAAAACTACAGAGCCCACCAAGCCCAATAACAGACAGAGTACA 9800

TCTCTGCAGCTTCACGACAAAGAGAACAATGGCGTCAAAGAAGGAGCCGTTGCAAAAGCAAAACGAGTGAGCTGTGCATCCGTATCAAAGATAAAGACGA 9900

AGAATCGATATGACGTTCTTAGCCTCCTCTCATCAAATGAATATGTGCGAAGGCTCCTAGGGTTTTCACAAGTGTATAAATAAAGAGACAGAGTCTCATT 10000

GTAAAAGTAAGAGAGAAGAGCTTAACAAAGTTTCAGTATTTCTCCTTCACTTTCAAATCTTTACAAAAAGTACACTATCACGTGAAATCATAAATCAAAC 10100

TGTTGCTGTGTAACGTCGTGAAAAAAAACGTTTTACAACAAAAAATAAGAATAGCGTCAACGCTAATTAAATTGCAGGTGGTCAACAATTGCGAGCAATT 10200

TACCGGGAAGAACAGACAAAGAAATAAAAAACTATTGGAATTCTCATCTCAGCCGTAAACTCCACGGTTACTTCAGAAAACCAACTGTCGTCAATACCGT 10300

CGAGAATGCGCCTCCGCCTCCTAAGCGTAGACCTGGAAGAACCAGCAGATCCGCCATGAAACCCAAATTTATCCTAAACCCTAAAAACCACAAAACCCCT 10400

AATTCTTTTAAAGCAAACAAAAGTGACATCGTTTTGCCAACTACGACAATAGAGAATGGAGAGGGAGACAAAGAAGACGCATTAATGGTGTTGTCAAGTA 10500

GTAGCTTAAGTGGAGCAGAGGAACCCGGTTTAGGACCATGTGGTTATGGAGACGATGGCGATTGTAACCCAAGCATTAATGGCGACGATGGAGCTTTGTG 10600

TCTCAATGACGACATTTTCGATTCTTGTTTTCTATTGGACGACTCTCATGCTGTCCACGTGTCCTCATGTGAGTCGAACAACGTAAAAAACTCTGAGCCA 10700

<-----AT3G62610_R1--

TATGGAGGGATGTCAGTTGGGCACAAAAATATCGAAACGATGGCTGATGATTTCGTTGACTGGGACTTTGTATGGAGAGAAGGTCAAACCCTTTGGGACG 10800

AAAAAGAGGATCTTGATTCGGTTTTGTCGAGGCTGTTAGATGGAGAGGAAATGGAATCTGAGATCAGACAAAGGGACTCCAACGACTTTGGAGAACCGTT 10900

GGATATTGACGAAGAAAACAAGATGGCTGCTTGCTTTGTCTTGTAA 10946
