# Supplementary material for: Identification of novel genes responsible for a pollen killer present in local natural populations of Arabidopsis thaliana
Source: PLoS Genet. 2025 Jan 13;21(1):e1011451. doi: 10.1371/journal.pgen.1011451 (PMC11761171; doi:10.1371/journal.pgen.1011451)
Supplement: S1 Fig — (PDF) [file pgen.1011451.s002.pdf]

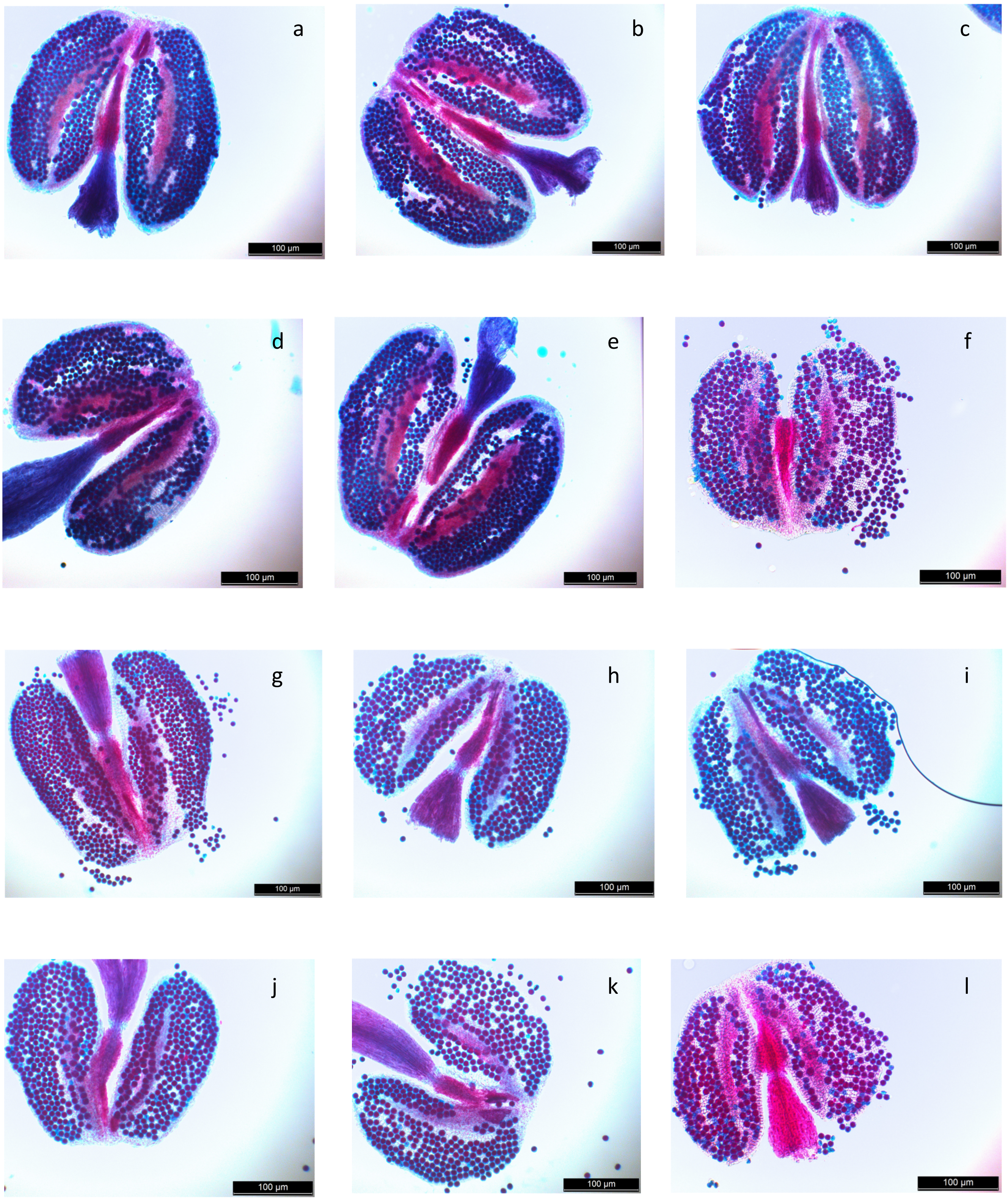

{MψFig: Pollen viability of mutants(Alexander coloration of anthers, viable pollen is colored in red and dead pollen appears blue).

Homozygous mutants *460*#1 (a) and *460*#2 (b) compared to the wild-type (c). Hybrids *460*#1 x Sha (d) and *460*#2 x Sha (e) compared to the control wild-type hybrid (f).

Homozygous mutants *apok3-like*#1 (g) and *apok3-like*#2 (h) compared to the wild-type (i). Hybrids *apok3-like*#1 x Sha (j) and *apok3-like*#2 x Sha (k) compared to the control wild-type hybrid (l).
