# Supplementary material for: Identification of novel genes responsible for a pollen killer present in local natural populations of Arabidopsis thaliana
Source: PLoS Genet. 2025 Jan 13;21(1):e1011451. doi: 10.1371/journal.pgen.1011451 (PMC11761171; doi:10.1371/journal.pgen.1011451)
Supplement: S2 Fig — (PDF) [file pgen.1011451.s003.pdf]

A

B

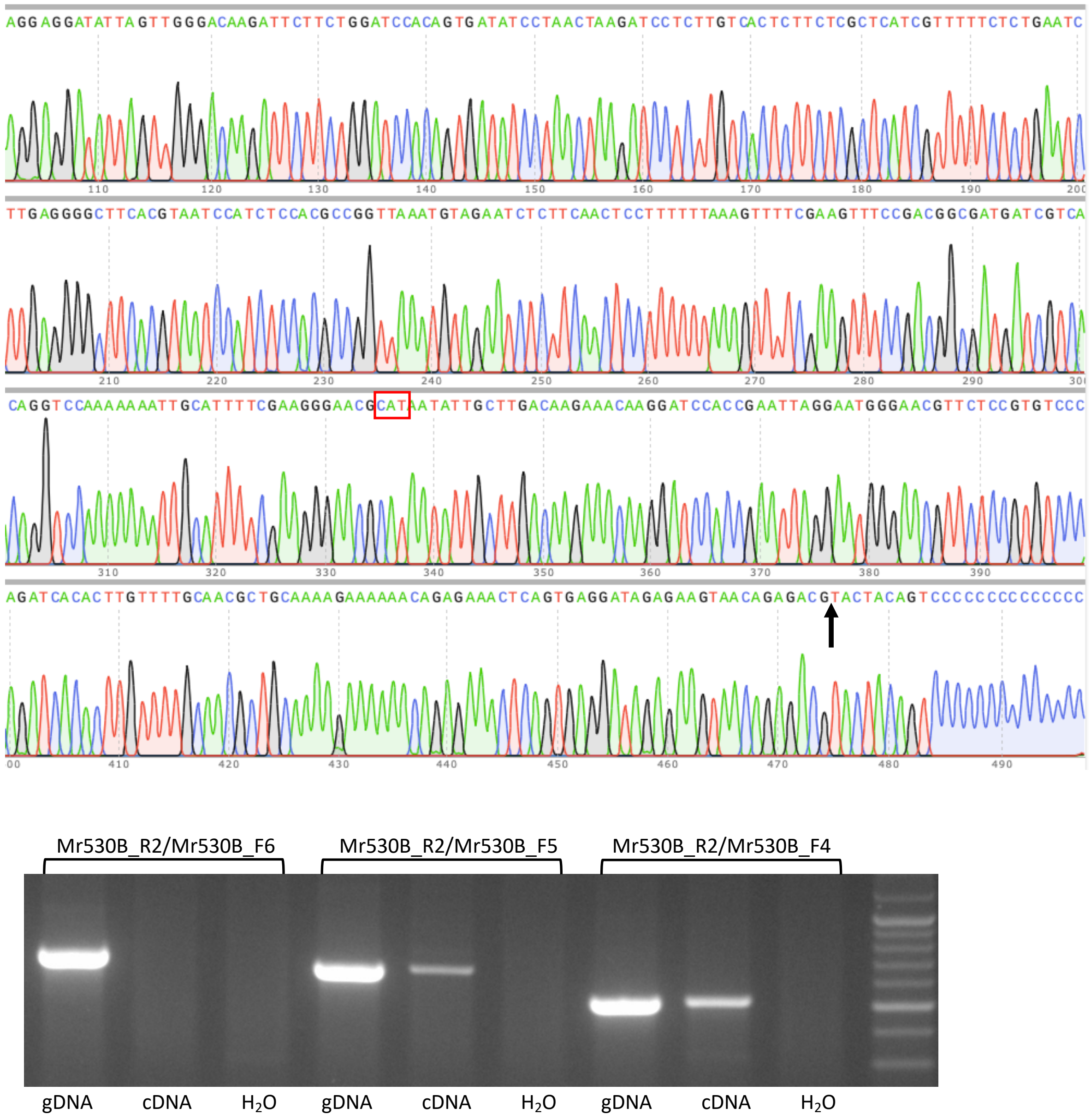

{HψFig: Validation of the *APOK3-like* (*KPOK3C*) gene structure predicted by EuGene.

A. Determination of the 5'-end of the transcript by sequencing the cDNA obtained by 5'-RACE with Mr530B_R3 (see File S; sequence is in reverse orientation). The start codon is framed in red and the 5'-end of the transcript predicted by Eugene is indicated by the black arrow.

B. RT-PCRs that confirm the absence of introns in the part of the gene which is not homologous to *APOK3*. gDNA: Mr-0 genomic DNA; cDNA: Mr-0 cDNA. See File S for the primers' positions on the sequence: Mr530B_F4 and Mr530B_F5 are located on *APOK3-lik*e 5'-UTR while Mr530B_F6 is located upstream. PCR Mr530B_R2/Mr530B_F6 serves as a control for the absence of genomic DNA in the cDNA.
