# Supplementary material for: Identification of novel genes responsible for a pollen killer present in local natural populations of Arabidopsis thaliana
Source: PLoS Genet. 2025 Jan 13;21(1):e1011451. doi: 10.1371/journal.pgen.1011451 (PMC11761171; doi:10.1371/journal.pgen.1011451)
Supplement: S3 Fig — (PDF) [file pgen.1011451.s004.pdf]

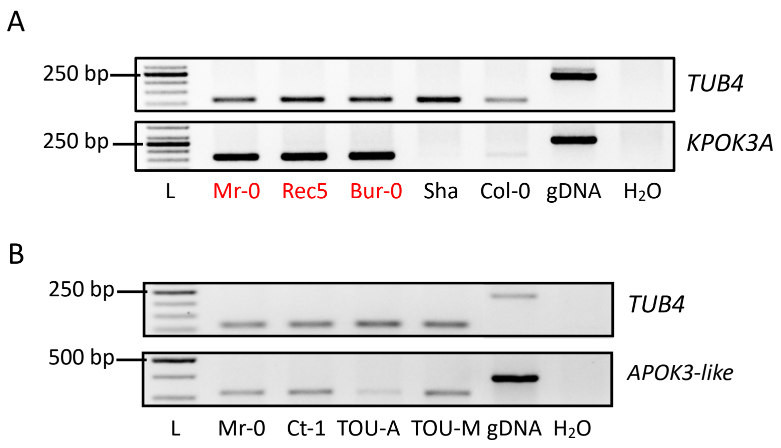

S3_Fig: Expression of *KPOK3A and KPOK3C* in leaves of diverse genotypes.

A. *KPOK3A* PCR amplification of cDNA from leaves of plants with different allelic forms of the gene (Mr-0 like, in red, or not, in black). cDNAs were synthetized from 0.2 µg of total RNA; 30 PCR cycles.

B. Expression of *KPOK3C*. *KPOK3C* PCR amplification of cDNA from leaves of different killer accessions. cDNAs were synthetized from 1 µg of total RNA; 28 PCR cycles for *TUB4* and 32 PCR cycles for *APOK3-like.* TOU-A: TOU-A1-111; TOU-M: TOU-M1-3.

TUB4 (*AT5G44340*) was used as control. gDNA: Mr-0 genomic DNA. L: GeneRuler 50 bp DNA Ladder (Thermo Scientific).
