# Supplementary material for: Identification of novel genes responsible for a pollen killer present in local natural populations of Arabidopsis thaliana
Source: PLoS Genet. 2025 Jan 13;21(1):e1011451. doi: 10.1371/journal.pgen.1011451 (PMC11761171; doi:10.1371/journal.pgen.1011451)
Supplement: S4 Fig — (PDF) [file pgen.1011451.s005.pdf]

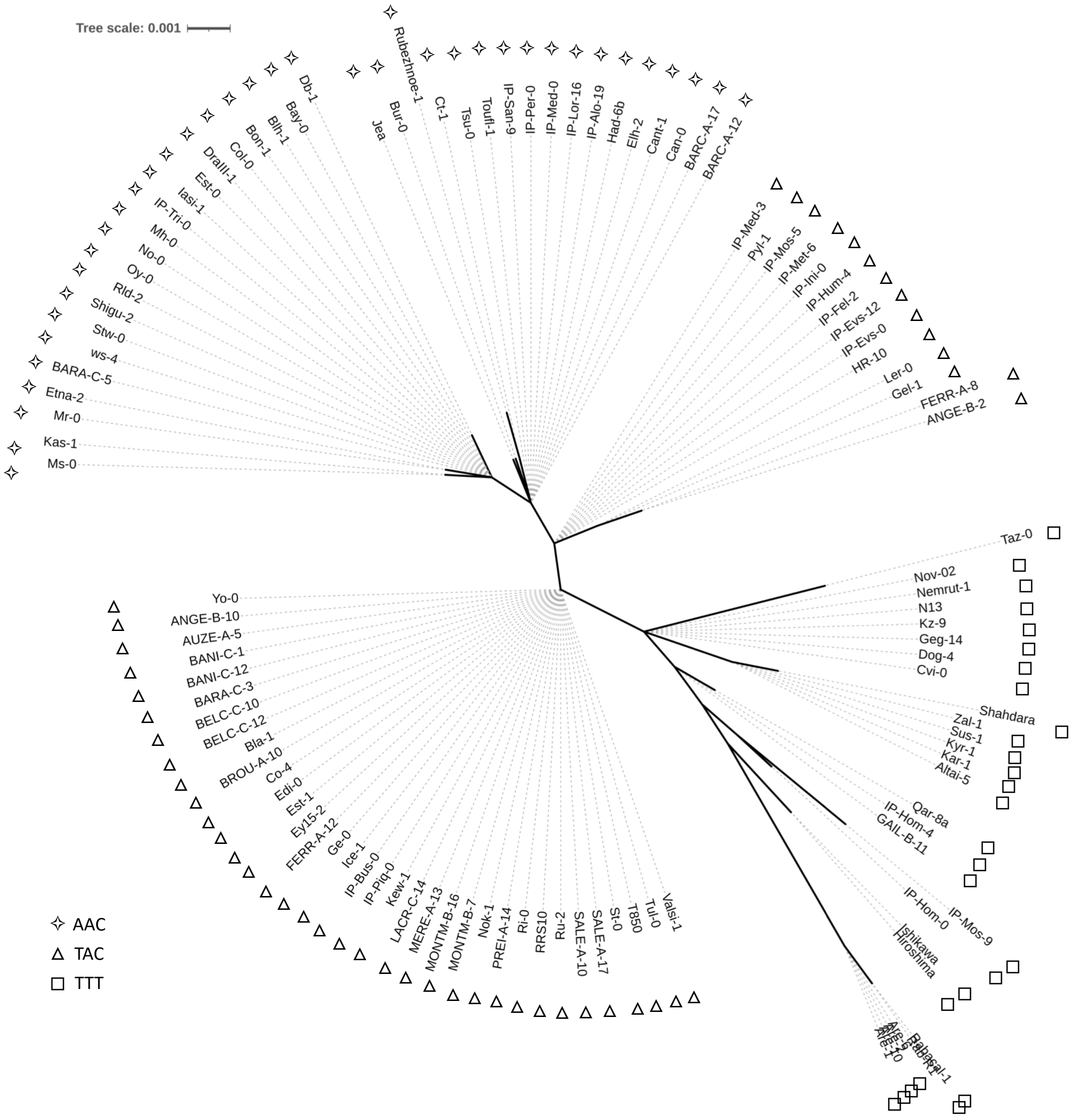

S4_Fig: Unrooted phylogenetic tree of *APOK3* sequences.

Nucleotidic sequences from 100 bp upstream of the ATG to the stop codon were used. For accessions with copies not perfectly identical, the most represented sequence was used. Symbols at the leaves indicate the fonctional type of the antidote, resistant (AAC), weak resistant (TAC), or sensitive (TTT).
