# Supplementary material for: Identification of novel genes responsible for a pollen killer present in local natural populations of Arabidopsis thaliana
Source: PLoS Genet. 2025 Jan 13;21(1):e1011451. doi: 10.1371/journal.pgen.1011451 (PMC11761171; doi:10.1371/journal.pgen.1011451)
Supplement: S5 Fig — (PDF) [file pgen.1011451.s006.pdf]

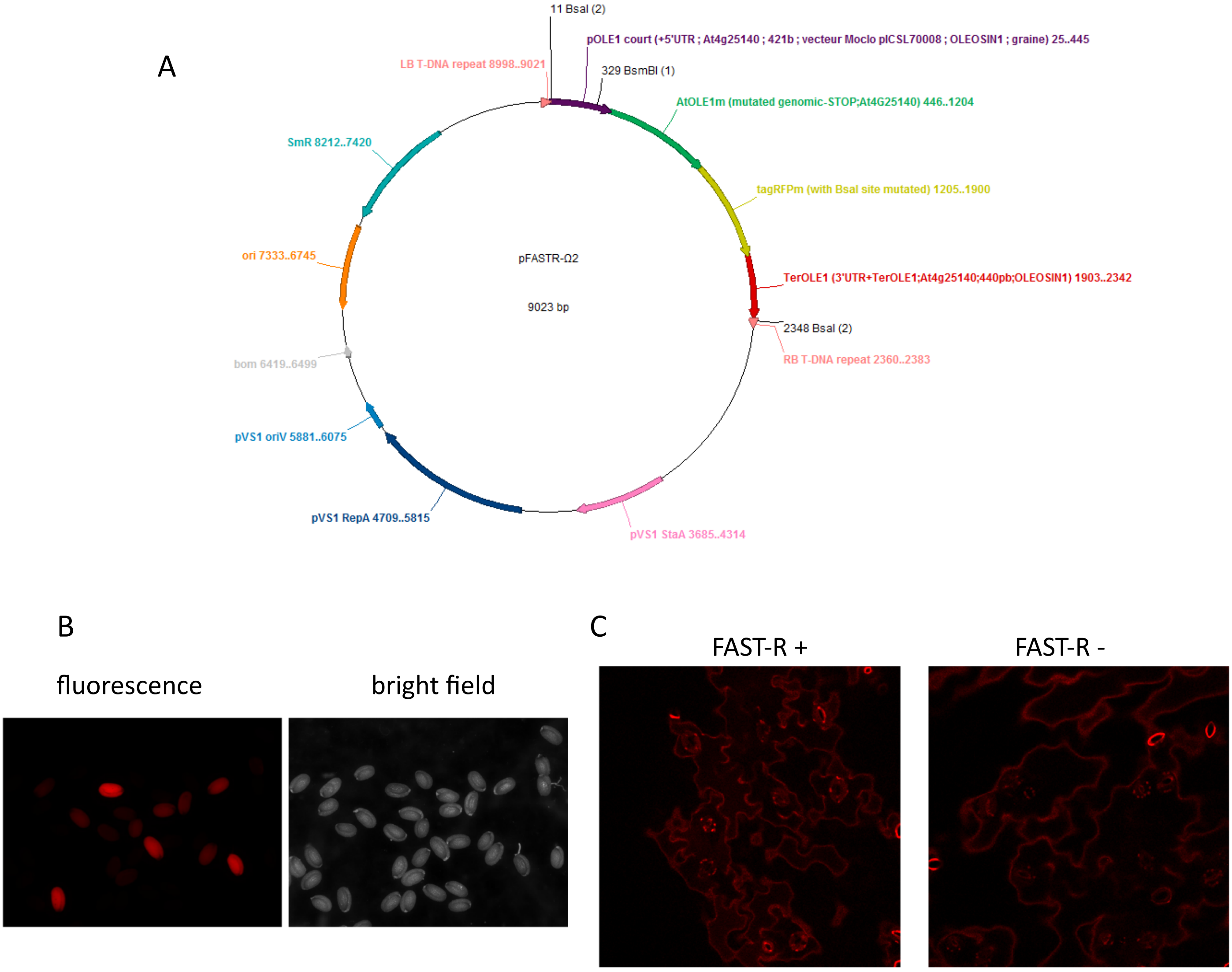

S5_Fig: **Use of the FAST-R seed-specific fluorescent marker for Arabidopsis transformant selection.** A. pFASTR-Ω2 binary vector map. The pFAST-R selection cassette from Moclo plants part kit (plasmid pICSL70008 in well E/11 of plate https://www.addgene.org/cloning/moclo/patron/#protocols-and-resources) was amplified and flanked in 5’ and 3’ by the prefixes 3+S1 GTCAGGAG and the suffix S8 CGCT, respectively, and by *Bsa*I restriction sites. The PCR fragment corresponding to the desired transcriptional unit (TU) was directly cloned into the destination vector pDGB3-Ω2 by *Bsa*I restriction/ligation reactions. B. Selection of red fluorescent transgenic seeds from infiltrated Arabidopsis plants under a fluorescence microscope. C. Typical confocal images of red fluorescence in leaf epidermis of 2 week-old T2 siblings carrying (left panel, FAST-R +) or not (right panel, FAST-R -). The respective genotypes were selected under the microscope as shown in B in order to check that the marker would not interfere with the cellular localization of other fluorescent proteins in leaf epidermis. The brightness and contrast were adjusted for better visibility of autofluorescence of stomata apertures and cell walls.
