# Supplementary material for: Identification of novel genes responsible for a pollen killer present in local natural populations of Arabidopsis thaliana
Source: PLoS Genet. 2025 Jan 13;21(1):e1011451. doi: 10.1371/journal.pgen.1011451 (PMC11761171; doi:10.1371/journal.pgen.1011451)
Supplement: Attachment — Submitted filename: ResponseToReviewers.docx [file pgen.1011451.s023.docx]

Dear Editor of *PLOS Genetics*,

Here is a revised version of the manuscript entitled ‘**Identification of novel genes responsible for a pollen killer present in Arabidopsis local natural populations**’, that we have submitted for publication in *PLOS Genetics* (PGENETICS-D-24-01160) - [EMID:2a310b1bf2683ae2]

We thank the three reviewers for their thorough and positive evaluation of our work and for their constructive comments. We respond below to each point raised by the reviewers. We have accepted all the reviewers' suggestions to improve the manuscript and make it easier to read. We hope that you will consider that the reviewers’ requests have been satisfied and that you will find that this revised version of the manuscript now fully meets PLOS Genetics's publication criteria.

Sincerely,

Christine Camilleri on behalf of all co-authors.

***Reviewer #1:*** *Ricou et al use a combination of genetic analyses, cell biology and population analyses to significantly advance understanding of the PK3 poison-antidote killer meiotic driver, previously discovered in the model plant A. thaliana. Previous work had identified the antidote of the system, APOK3 and showed that two regions (A and C) flanking APOK3 are required for killing. This work demonstrated that one copy of APOK3 can be sufficient to provide the antidote function and identified a novel APOK3 allele that is weakly resistant to the driver’s poisons. This work also identifies two poison genes, KPOK3A and KPOK3C, that are each necessary for the pollen killing. The authors were also able to show that KPOK3A is the only gene required for the killing in the ‘A’ region. Similar experiments to test if KPOK3C was the only killer in the ‘C’ region failed, but were informative in they suggest specific control of KPOK3C expression is important. The authors broadly sampled A. thaliana isolates and identified several alleles of both killer genes. They also analyzed haplotype frequencies and characterized haplotype phenotypes via crosses. Finally, they provided phylogenetic analyses of the three genes to provide insight on their evolutionary histories. Finding killer and sensitive haplotypes within populations is especially exciting. This is a strong paper, and I enjoyed reading it. With one exception, noted below, the claims were sufficiently supported by the data. My suggestions for the authors to consider are listed below.

Major suggestion:
The abstract claims the data ‘indicates that the pollen killer activity involves the mitochondria.’ I suggest rewording that claim as the molecular mechanisms of the killing are still unclear. This could be as easy as saying the data ‘suggest’ rather than ‘indicates.’ I agree that mitochondrial localization and phenotypic changes depending on the cytoplasmic background of the hybrids are both consistent with mitochondrial function, but more is required to conclusively demonstrate it.*

We replaced ‘indicates’ with ‘suggests’ in the abstract.

*I also think it is important to state: 1) if you tested the phenotype of the tagged genes to see if they remained active*

The tagged genes were under the control of the *UBQ10* promoter, which was found not to be expressed in meiocytes (see figure below). We were unable to complement the recombinants or the mutants with the genes under the control of this promoter even without the tag, so we have not tested the tagged constructs.

Figure: Immature anther at the meiocyte stage (prophase) from a plant expressing the KPOK3C-citrine fusion protein under the control of the pUBQ10 promoter. The 'empty' zone in the left image corresponds to the meiocytes (author Laurence Cromer).

*and 2) if you performed western blots to see if the fluorescent proteins were staying attached to full length proteins. In my own experience, I have had tags disrupt function and had tags be clipped from proteins and end up in mitochondria.*

We agree that the localization of KPOK3C in particular has to be more thoroughly investigated, and this is discussed in the paper. Further investigation of the localization of the pollen killer proteins should involve biochemical experiments, including western blots, but we think this is beyond the scope of this study.
*Minor suggestions/comments:
The readability of the paper could be significantly improved by adding more extensive labeling to the figures. I could always find the information I needed to fully understand the figures by reading the legends. Still, I found myself adding guiding texts and labels to several figures, so I did not have to keep reading the figure legend each time I returned to a figure. I will list the additions I made to my copy of the figures; in case the authors decide to add some of these things to help guide future readers.
-There are several distinct things illustrated in panel 2A. Perhaps break into more panels (e.g. one for KPOK3A and 3C). It is also unclear where the presented KPOK3C sequence is relative to the gene cartoon.*
We separated panel 2A in two panels, one for *KPOK3A* and one for *KPOK3C*. And we indicated the position of the presented *KPOK3C* sequences on the gene cartoon.

*-Figure 2B and other such plots: More complete genotype labels on the X-axes and formal labels on the Y-axes (e.g. % homozygotes in F2 progeny).*

This was done (also on Figures 3F, 5B, 6 and 11).
*-Figure 3D: The labels 1 and 2 could be replaced with more informative labels*

This was done.
*-Figure 4, 8, 10 (the pinheads) and 11: A key for the color coding would be helpful.*

This was done on Figure 8. For the others, we left the information in the legend to avoid overloading the figure.
*-Figure 9: I think this got distorted in PDF production, but the colors are not very easy to distinguish.*

We now use a better image quality. The palette is color-blind friendly, and dots in the inset were enlarged for better readability.
*Many mutations are described as knock-outs, but all seem to leave coding sequence intact, leaving open the door for remaining gene function.*

The mutants described as knock-outs had frameshifts from the targeted mutation points, which result in proteins whose sequences are largely altered, and shorter than the wild-type (more details have been added in the legend of Fig 2). In addition, the phenotype of these mutants showed that the resulting proteins were non-functional for the pollen killer activity.

The mutant *kpok3c*#3 was not described as a knock-out because it only had one amino-acid deletion and one amino-acid change (although the killer activity of *KPOK3C* was in fact found to be inactivated in this genotype).
*Are the oligos used in 3B on spots that are invariant?*

Yes, they are. This was added in the figure legend: “The primers used were defined on sequences that were invariant between the genotypes tested.”
*Line 143: were these gene introductions random, or targeted in some way?*
They were random, because the genes were introduced into the plants by *Agrobacterium* *tumefaciens* transformation.

*More information in the text/figure legend about what tissue is shown in Figure 4 is required.*
It was in the “Material and Methods” section. We also added this in the figure legend.
*Line 217: I was confused by ‘PK3 locus against the Mr-0 allele’ as I was thinking of the wt Mr-0 allele. This could be avoided by replacing ‘Mr-0 allele’ with ‘double mutant allele.’*
This was done.
*Line 255: I think the table S1 should be S6.*

That’s right, it was corrected.
*Table 3: It would be great to include the % sha homozygotes as a column in this table. The new data from this paper is in S6, but it would be nice to see it all together.*
This was done.
*Line 380: The t-haplotype in mouse has more than one killer gene. I think those act additively, rather than co-dependently.*
“To our knowledge, only two gamete killers with more than one killer gene have been reported so far”: we added “in plants” (now line 397).
*Line 515: Might those be due to non-allelic gene conversion, a major driver of WTF gene evolution?*

This is an interesting hypothesis. However, it would be difficult to test because the sequences of the *APOK3* and *KPOK3C* present on killer alleles present very few polymorphisms. We have also corrected a remaining error in the pointed sentence (now line 532).
*The framing of your work in the introduction and discussion was very nice.
The use of red and black type within the some of the figures to distinguish killer and sensitive types was very helpful.
The supplemental data tables were nicely organized and easy to navigate.*

***Reviewer #2****: The manuscript “Identification of Novel Genes Responsible for a Pollen Killer in Local Natural Populations of Arabidopsis thaliana” by Ricou et al. investigates pollen killer genes using both global accessions and local populations of A. thaliana. The manuscript presents and discusses results in a clear, logical, and well-written manner, with high-quality figures throughout. In this study, the authors identified killer components within the previously known pollen killer locus, PK3. Leveraging available sequence data for this killer element, they found evidence suggesting its association with mitochondria. They also confirmed that killer activity was pollen-specific and, through extensive sequence analysis, identified distinct haplotypes for the killer locus, enabling them to identify additional killer accessions. Interestingly, they discovered that killer and non-killer haplotypes co-exist in natural populations, raising questions about why these killer alleles persist in nature and whether they might confer benefits under certain backgrounds or conditions. This paper contains a substantial amount of well-presented data that was highly engaging to read.*

*I only have a few minor suggestions:
In the Introduction, lines 69-87 could be revised to clarify which results are previously established findings and which are novel contributions from this study.* *Additionally, referencing result figures in the Introduction may not be common practice and could be reconsidered.*

We have clarified the text. Figure 1 is very helpful for visualizing the locus complexity, and we reference it in the introduction precisely because it shows previously established results (Simon et al 2022).
*Figure 2: For clarity in point B, it would help to label the control as F1 (or is it F2?). Also, please add a y-axis label to indicate what the percentage represents. The same applies to Fig.* *3B* (actually 3F)*, Fig. 5B, and Fig. 6; please add y-axis labels to these figures for consistency.*
This was done (with the addition of more complete genotype labels on the x-axes).
*Figure 7B: Could you specify what "frequency" refers to in this figure?*

We made this clearer in the figure and the legend.

*Additionally, it would be helpful to explain how the sum of frequencies could exceed 100%.*

We are sorry, this was a mistake on our part. The figures have been corrected and the sum is now 100% (they were correct in the text, lines 328 and 330-331). Thank you for your vigilance and for pointing this out so kindly!
*Lastly, regarding the wild populations, since no resistant types were observed, can it be ruled out that such resistance alleles might be deleterious or, if pollen-specific, would not lead to seed production?*

The resistant allele is rather widespread in the sample of worldwide accessions (32% of 728 accessions). Yet, we cannot formally rule out that it might be deleterious. It would be necessary to analyze the evolution of this allele frequency in wild populations where it exists, such as the CON and ETA-2 populations (the individuals Con-1 and Eta2-1 have resistant alleles, see Table S7).

*Was the analysis of local populations based on tissues collected directly from the wild, or were seeds collected from the wild and then grown in the greenhouse? This information is currently not specified in the Materials and Methods section.*

Seeds were collected from the wild and then grown in the greenhouse. This information was added to the Materials and Methods section.

***Reviewer #3:*** *In their manuscript “Identification of novel genes responsible for a pollen killer present in local natural populations of Arabidopsis thaliana” the authors first identify 2 “killer” genes involved in a 3-gene poison-antidote system that naturally segregates in the model system Arabidopsis thaliana, and for which they have already identified the antidote. They find that these two genes border the antidote and are tightly genetically linked, as would be expected by theory. They then go on to describe the cellular localization/targets of the poison (and antidote), finding that they co-localize to the mitochondria. They then describe the global distribution of this system, finding incredible polymorphism and that both a the haplotype that lacks the antidote and the killer haplotype have likely descended from a COL-0 like allele that possess the antidote, but lacks the killer genes, providing evidence for an “antidote first” model. Lastly, they discover that killer/resistant/sensitive alleles segregate on fine spatial scales (i.e. within populations), making it a potentially active killer-antidote system in nature, and describe a range of killer-like and resistant-like behaviors in lines from the same population.
The manuscript is a tour de force, genetically speaking, and in my opinion has gone above and beyond to rigorously describe an exciting system which will interest both evolutionary biologists and molecular geneticists alike. I offer only a handful of very minor suggestions. Congrats to the authors!

Minor
- There are a few locations where Arabidopsis is not italicized throughout the manuscript, so should be double checked.*

We used the convention where Arabidopsis is italicized when it is part of the Latin name *Arabidopsis thaliana*, whereas it is not when used alone as a common name specifically

for *A. thaliana*. (Other genus names often used in this manner, as a common name for a particular species, for example Chlamydomonas for Chlamydomonas reinhardtii and Drosophila for Drosophila melanogaster.)

*- Line 25: grammatically, I think the sentence should read either “…in local populations remains…” or “…in a local population remains…”*

We changed it for “…in local populations remains…”

*- Line 54: the word “favoring” suggests will/intention. I would replace with “resulting in” or similar.*

We replaced “favoring” with “which can lead to”.
*- Line 270- the authors state that they cannot access the 1001 genomes because they have been aligned to Col-0. Is it not possible to access the original fastq files and align them to one of the newer de novo genomes from Lian et al. 2024. Nature Genetics in which this region is present?*

Indeed, it would be theoretically possible to align the 1001 Genomes sequences to the *de novo* sequence of a killer genotype. The problem is that the locus is subject to such important structural variations, particularly in killer genotypes (see Figure 1, and for more genotypes Figure 5 from Simon et al. 2022), that it would be very difficult to choose the genome on which to align the others. In addition, the killer genotypes contain at least two or three copies of *APOK3*, plus one copy of the homologous region of *KPOK3C*, which could hamper the proper alignment.

*- Line 537: I don’t think “coherent” is the correct word- “consistent”, perhaps?*

We replaced “coherent” with “consistent”.
*- Figure 9 is very blurry and hard to read, and I recommend the authors use a higher image quality in the final version.*

The figure should now be easily readable.
